# Usefulness of circuit training at home for improving bone mass and muscle mass while losing fat mass in undergraduate female students

**DOI:** 10.1186/s12944-018-0743-3

**Published:** 2018-05-09

**Authors:** Yoko Takahata

**Affiliations:** Faculty of Nursing, Baika Women’s University, Shukunosho 2-19-5, Ibaraki, Osaka 567-8578 Japan

**Keywords:** Undergraduate female students, Calcaneus QUS status, Circuit training, Ultrasound bone densitometer, Body composition

## Abstract

**Background:**

The purpose of this study was to determine whether or not circuit training at home affects the calcaneus quantitative ultrasound status as well as other indices of body composition among undergraduate female students.

**Methods:**

Forty-one adolescents were recruited (18.5 ± 0.6 years old). The stiffness index of the calcaneus, broadband ultrasound attenuation of the calcaneus, speed of sound of the calcaneus, and body frame index. This was a three-month intervention study, so the measurements were conducted at baseline, 2 months later, and 3 months later while the subjects underwent circuit training at home. The subjects were divided into two groups: namely, the exercising group and non-exercising group.

**Results:**

In the exercising group, broadband ultrasound attenuation of the calcaneus was higher 2 months later (*p* = 0.033) as well as 3 months later (*p* = 0.036), and the speed of sound of the calcaneus was higher 3 months later (*p* = 0.018). In addition, the muscle mass was strongly positively correlated with the calcaneus QUS-SOS (*p* = 0.004), while the body fat percentage was a strongly negatively correlated with the calcaneus QUS-BUA (*p* = 0.043). In the non-exercising group, the stiffness index of the calcaneus was higher 2 months later (*p* = 0.002) as well as 3 months later (p = 0.002). Furthermore, the body percentage was strongly positively correlated with the calcaneus QUS-SI (*p* = 0.009).

**Conclusions:**

These findings suggest that the calcaneus quantitative ultrasound status and muscle mass while losing fat mass may be improved by means of a simple exercise regimen within a short period among undergraduate female students.

## Background

It is important to attain a sufficient peak bone mass and reduce the risk of osteoporosis in later life, as the levels of biochemical bone turnover markers peak in the middle of puberty and begin to decrease in late puberty, according to, Harel et al., Walsh et al., and Yilmaz et al. [1–3]. In addition, Walsh et al. mentioned the bone turnover cycle as being related to the gonadal hormones, especially estrogen in women [2], and a sex steroid hormone-dependent increase in the longitudinal growth and periosteal bone expansion for pubertal girls was observed by Libanati et al. [4]. A sufficient supply of sex steroid hormones, such as that achieved with regular menstruation, is important for sustaining bone mass, as osteoporosis or osteopenia can easily develop in postmenopausal women [5].

There has been some discussion about the relationship between exercise and bone mass [6–11]. These previous studies focused on the potential for high-impact load exercises to increase the bone mass [7, 10] and the risk of overstress causing bone fracture [11]. Efficient muscle strength also affects the bone mass, even in females [9, 10]. According to Carrasco et al., Leppänen et al. and Villareal et al., even elderly people can increase their muscle mass and strength and the functional level of their body by sufficient exercise, which subsequently affects the bone mass [12–14].

In general, it is well known that physical activities prevent chronic diseases and reduce the risk of premature death. It is important to start good exercise habits at a young age [15]. Recently, sarcopenic obesity has been increasingly recognized and therefore it is important to develop strategies for reducing the risk of developing sarcopenic obesity is to maintain sufficient muscle mass [16]. While there are many ways to exercise, circuit training was selected for focus in the present study, as both anaerobic and aerobic exercise can be performed continuously for a certain period of time. This workout seems to affect the muscle strength even if the workout level is a low-intensity one [17]. The effect is not obvious if the patients have knee problems, such as in individuals who have undergone arthroplasty [18]. The muscle mass or strength is one factor affecting the bone mass, and ideally, exercise should be relatively easy to continue, as older people have been subjects in previous studies. Many undergraduate students stop exercising after entering university and do not seem to exercise often. For this reason, I chose an exercise that was effective and not very difficult to continue.

In the present study, I examined whether or not circuit training at home affects the calcaneus quantitative ultrasound (QUS) index as well as other indices of body composition among undergraduate female students.

## Methods

### Subjects

A total of 41 healthy, untrained undergraduate female students (18.5 ± 0.6 years old) were recruited for the study by poster advertisement and a short presentation in classes. Those who wanted to participate contacted me by e-mail. I first confirmed that all of the subjects were in good health and were free of chronic diseases affecting their bone metabolism. Informed consent was obtained from all of the subjects at an initial investigation in accordance with the Declaration of Helsinki. The study was approved by the ethical review boards at the authors’ institution.

### Study design

This was three-month intervention study. The initial investigation day was scheduled for each subject to explain the protocol in detail in order to confirm they had given their informed consent. After obtaining their informed consent, I distributed a self-reporting questionnaire and took several body measurements at the initial day. These measurements were repeated 2 and 3 months later as well.

### Experimental procedures

The speed of sound (SOS), broadband ultrasound attenuation (BUA), and stiffness index of the calcaneus (SI) were measured using an ultrasound bone densitometer (Achilles InSight; GE Healthcare, Little Chalfont, UK). I used an ultrasound bone densitometer because it has no side effects and correlates well with the dual-energy X-ray absorptiometry (DXA)-measured bone mineral density (BMD) [19–24] or bone mineral contents (BMC) [24] as well as quantitative computed tomography (QCT) [25]. The densitometer was operated by a researcher experienced with taking measurements, and the final intra-sample mean coefficient of variation for the stiffness index was 0.7%.

The subjects’ height (determined with a DST-210 N; Muratec-KDS Corp., Kyoto, Japan) and weight, body fat percentage, and muscle mass (determined with a DC-320; Tanita Corp., Tokyo, Japan) were measured after shoes had been removed, and then the body mass index (BMI) was calculated.

### Questionnaire

Specific details asked for in the self-reporting questionnaire included birthday, secondary sexual characteristics, exercise habits in junior high school, and exercise habits in high school. The age of onset of secondary sexual characteristics was determined based on the age at which menstruation started.

### Self-made circuit training

Circuit training involves performing both anaerobic and aerobic exercise continuously for a certain period of time. While there are numerous training gyms which can be used for circuit training in Japan, it was not realistic to ask subjects to go to the gym at least three times a week or prepare an environment for training at home. I selected anaerobic and aerobic exercises that did not require any tools or a huge space, and on the initial day of the investigation, I showed the subjects a list of the exercises so that they could choose and structure own exercise schedules. The anaerobic exercises included “sit-ups”, “squats”, “push-ups”, “calf raising and lowering movements”, and “hip raising and lowering movements” and the aerobic exercises included “step aerobics”, “shadow boxing”, and “jogging in place”. Every subject wrote down the exercise name on their sheet as an anaerobic or aerobic exercise on the sheet (Box 1).

### Statistical analyses

An unpaired *t*-test was used to determine if the body frame changed over time among the total subjects as well as to determine whether or not any specific factors in the body frame at baseline differed between the subjects performed the circuit training for the first 2 months and those not exercising. A paired *t*-test was used to determine how the body frame factors changed in the two groups who performed the circuit training and those who did not. A multiple regression analysis was also performed to determine the effect of muscle mass or body fat percentage on calcaneus QUS-SOS, calcaneus QUS-BUA, or calcaneus QUS-SI. A *p* value of < 0.05 was considered to be statistically significant. Statistical analyses were performed using the IBM SPSS 23.0 software program (IBM Corp. Released 2015. IBM SPSS Statistics for Windows, Version 23.0. Armonk, NY USA.).

## Results

### Subjects’ characteristics

The subjects’ characteristics are listed in Table 1. Among all subjects, most factors (except for the calcaneus QUS-BUA) did not change significantly between the baseline and 3 months later.

**Table 1:** Characteristics of total subjects, *n* = 41

	Factors	Means ± SD (range)	t-score^b^
	Age (years)	18.5 ± 0.6 (18–20)	
	Height (cm)	157.7 ± 5.2 (145.6–167.4)	
	Secondary sexual characteristic development (age in years)^a^	12.1 ± 1.4 (9.0–16.0)	
Measurement at the baseline	Calcaneus QUS-SI (Stiffness index)	104.7 ± 16.2 (75.0–151.0)	
	Calcaneus QUS-SOS (m/s)	217.7 ± 54.6 (91.0–255.0)	
	Calcaneus QUS-BUA (dB/MHz)	33.1 ± 2.6 (31.0–41.0)	
	Weight (kg)	51.2 ± 7.0 (38.9–73.5)	
	Body Fat Percentage (%)	27.7 ± 4.6 (17.4–39.3)	
	BMI (kg/m^2^)	20.6 ± 2.5 (15.0–28.6)	
	Muscle mass (kg)	34.7 ± 3.1 (28.3–43.0)	
Measurement after 2 months	Calcaneus QUS-SI (Stiffness index)	109.5 ± 18.2 (74.0–163.0)	1.075
	Calcaneus QUS-SOS (m/s)	212.4 ± 53.3 (93.0–255.0)	0.658
	Calcaneus QUS-BUA (dB/MHz)	32.1 ± 0.6 (29.0–33.0)	1.877
	Weight (kg)	51.4 ± 7.0 (40.4–73.3)	0.117
	Body Fat Percentage (%)	28.1 ± 4.6 (19.7–39.1)	0.427
	BMI (kg/m^2^)	20.7 ± 2.4 (15.6–28.5)	0.200
	Muscle mass (kg)	34.7 ± 3.0 (28.0–42.9)	0.530
Measurement after 3 months	Calcaneus QUS-SI (Stiffness index)	108.6 ± 16.7 (74.0–166.0)	1.264
	Calcaneus QUS-SOS (m/s)	221.1 ± 51.8 (80.0–255.0)	0.839
	Calcaneus QUS-BUA (dB/MHz)	32.4 ± 0.7 (31.0–34.0)	2.644 ^†^
	Weight (kg)	51.2 ± 7.1 (40.0–74.2)	0.038
	Body Fat Percentage (%)	28.5 ± 4.7 (18.7–40.1)	0.760
	BMI (kg/m^2^)	20.6 ± 2.5 (15.4–28.9)	0.013
	Muscle mass (kg)	34.3 ± 2.9 (28.3–42.0)	0.633

*2D:4D* ratio of the finger length of the 2nd and 4th digit, *QUS-SI* stiffness index by quantitative ultrasound, *QUS-SOS* speed of sound by quantitative ultrasound, *QUS-BUA* broadband ultrasound attenuation by quantitative ultrasound, *BMI* body mass index

^a^The age at which menstruation started

^b^t-score by an unpaired *t*-test (two-sided test) showed the differences between the baseline and either 2 or 3 months after baseline

[†] indicates a significant relationship between the baseline and the time of follow-up; † < 0.05

### Differences in the body frame index at the baseline between the exercising and non-exercising groups

The differences in the body frame index at the baseline between the exercising and non-exercising groups are shown in Table 2. There were no significant relationships in any factors between the two groups. However, most of the factors in the exercising group were slightly higher than those in the non-exercising group, except for the calcaneus QUS-SI and calcaneus QUS-BUA.

**Table 2:** The differences in the body frame index at the baseline between the exercising and non-exercising groups by an unpaired *t*-test (two-sided test)

Factors	Subjects		*p*
	Exercising group^*1^ (*n* = 22)	Non-exercising group^*2^ (*n* = 19)	
Weight (kg)	50.13 ± 7.05	52.52 ± 6.89	0.280
Body Fat Percentage (%)	27.30 ± 4.70	28.13 ± 4.65	0.576
BMI (kg/m^2^)	20.24 ± 2.44	21.01 ± 2.46	0.319
Muscle mass (kg)	33.87 ± 2.95	34.86 ± 2.94	0.292
Calcaneus QUS-SI	105.50 ± 15.23	103.79 ± 17.61	0.740
Calcaneus QUS-SOS (m/s)	214.05 ± 61.51	225.11 ± 45.97	0.524
Calcaneus QUS-BUA (dB/MHz)	33.36 ± 3.00	32.95 ± 2.04	0.612

*BMI* body mass index, *QUS-SI* stiffness index by quantitative ultrasound, *QUS-SOS* speed of sound by quantitative ultrasound, *QUS-BUA* broadband ultrasound attenuation by quantitative ultrasound

^*1^: Subjects performed the circuit training at home at least 3 times a week for the first 2 months

^*2^: Subjects did not perform the circuit training

### Differences in the body frame index at two and three months later between the exercising and non-exercising groups

How the body frame index changed at 2 and 3 months later in both the exercising and non-exercising groups is shown in Table 3.

**Table 3:** The differences in the body frame index at two and three months later between the exercising and non-exercising groups by a paired *t*-test (two-sided test)

	Baseline		2 months later				3 months later			
	M	SD	M	SD	t score	*p* values	M	SD	t score	*p* values
Exercising group, *n*=22										
Weight (kg)	50.1	7.05	50.2	7.11	0.11	0.91	50.1	7.12	0.15	0.89
Body Fat Percentage (%)	27.3	4.70	27.4	4.75	0.25	0.80	27.9	4.86	2.06	0.53
BMI (kg/m^2^)	20.2	2.44	20.3	2.40	0.11	0.91	20.2	2.47	0.16	0.88
Muscle mass (kg)	33.9	2.95	34.2	2.96	2.59	*0.017*	34.2	2.95	2.36	*0.028*
Calcaneus QUS-SI	105.5	15.2	108.9	17.5	1.36	0.19	110.8	18.2	2.07	0.05
Calcaneus QUS-SOS (m/s)	206.0	60.6	214.1	61.5	0.93	0.37	231.1	45.0	2.56	*0.018*
Calcaneus QUS-BUA (dB/MHz)	32.0	0.8	33.4	3.0	2.28	*0.033*	32.4	0.7	2.25	*0.036*
Non-exercising group, *n*=19										
Weight (kg)	52.5	6.89	52.9	6.84	1.66	0.11	52.4	6.98	0.35	0.73
Body Fat Percentage (%)	28.1	4.65	29.0	4.30	2.52	*0.021*	29.1	4.61	3.36	*0.003*
BMI*^1^ (kg/m^2^)	21.0	2.46	21.2	2.37	2.66	*0.016*	21.0	2.47	0.32	0.75
Muscle mass (kg)	35.4	3.13	35.3	3.04	0.70	0.49	34.9	2.94	3.38	*0.003*
Calcaneus QUS-SI	103.8	17.6	108.0	18.6	3.66	*0.002*	108.3	16.2	3.54	*0.002*
Calcaneus QUS-SOS (m/s)	209.5	57.8	217.5	44.8	1.09	0.292	225.1	46.0	1.99	0.062
Calcaneus QUS-BUA (dB/MHz)	32.2	0.50	33.0	2.04	1.76	0.096	32.4	0.68	1.48	0.157

*BMI* body mass index, *QUS-SI* stiffness index by quantitative ultrasound, *QUS-SOS* speed of sound by quantitative ultrasound, *QUS-BUA* broadband ultrasound attenuation by quantitative ultrasound

Exercising group, *n* = 22

Non-exercising group, *n* = 19

The numerical value in italicized face indicates a significantly higher than the value at baseline

In the exercising group, the subjects attained a significantly higher muscle mass (*t* = 2.59, *p* = 0.017) and calcaneus QUS-BUA (*t* = 2.28, *p* = 0.033) at 2 months later and muscle mass (*t* = 2.36, *p* = 0.028), calcaneus QUS-SOS (*t* = 2.56, *p* = 0.018), and calcaneus QUS-BUA (*t* = 2.25, *p* = 0.036) at 3 months later from the baseline. The score of calcaneus QUS-SOS at 2 months later did not significantly differ from that at baseline, although some increase was noted. Similarly, the scores of calcaneus QUS-SI at 2 and 3 months later did not significantly differ from that at baseline, although some increase was noted. Multiple regression models showed about 21% of the variance of calcaneus QUS-SI, 18% of the variance of calcaneus QUS-SOS, and 28% of the variance of calcaneus QUS-BUA in the exercising group. The results showed a positive effect of muscle mass on the calcaneus QUS-BUA (*p* = 0.054), a negative effect of body fat percentage on the QUS-BUA (*p* = 0.043) and a positive effect of body fat percentage on the calcaneus QUS-SOS (*p* = 0.004) 3 months later, shown in Table 4.

**Table 4:** The effect of muscle mass or body fat percentage on calcaneus QUS status in both exercising group and non-exercising group by a multiple regression analysis

Exercising group, *n* = 22				
		Factors	β	*p* values
Model I	Calcaneus QUS-SI	Body Fat Percentage (%)	-0.129	0.768
		Muscle mass (kg)	0.303	0.061
Model II	Calcaneus QUS-SOS (m/s)	Body Fat Percentage (%)	- 0.126	0.627
		Muscle mass (kg)	0.902	*0.004*
Model III	Calcaneus QUS-BUA (dB/MHz)	Body Fat Percentage (%)	- 0.577	*0.043*
		Muscle mass (kg)	0.477	0.054
Model I: Adjusted R^2^=0.21 (*p* < 0.001), Model II: Adjusted R^2^=0.18 (*p* < 0.001), Model III: Adjusted R^2^=0.28 (*p* < 0.001)				
Non-exercising group, *n* = 19				
		Factors	β	*p* values
Model I	Calcaneus QUS-SI	Body Fat Percentage (%)	0.847	*0.009*
		Muscle mass (kg)	0.110	0.700
Model II	Calcaneus QUS-SOS (m/s)	Body Fat Percentage (%)	0.759	0.051
		Muscle mass (kg)	0.360	0.071
Model III	Calcaneus QUS-BUA (dB/MHz)	Body Fat Percentage (%)	0.639	0.060
		Muscle mass (kg)	- 0.399	0.089
Model I: Adjusted R^2^=0.22 (*p* < 0.001), Model II: Adjusted R^2^=0.17 (*p* < 0.001), Model III: Adjusted R^2^=0.20 (*p* < 0.001)				

QUS-SI, stiffness index by quantitative ultrasound; QUS-SOS, speed of sound by quantitative ultrasound

QUS-BUA, broadband ultrasound attenuation by quantitative ultrasound

The numerical value in italicized face indicates a significant correlative factor of each calcaneus QUS status

In the non-exercising group, the subjects attained a significantly higher body fat percentage (*t* = 2.52, *p* = 0.021), BMI (*t* = 2.66, *p* = 0.016), and calcaneus QUS-SI (*t* = 3.66, *p* = 0.002) at 2 months from baseline and significantly higher body fat percentage (*t* = 3.36, *p* = 0.003) and calcaneus QUS-SI and significantly lower muscle strength (*t* = 3.38, p = 0.003) at 3 months from baseline. The scores of calcaneus QUS-SOS at two and 3 months later did not significantly differ from that at baseline, although some increase was noted. The multiple regression models showed about 22% of the variance of calcaneus QUS-SI, 17% of the variance of calcaneus QUS-SOS, and 20% of the variance of calcaneus QUS-BUA in the non-exercising group. The results showed a positive effect of body fat percentage on the calcaneus QUS-SI (*p* = 0.009) 3 months later, also shown in Table 4.

## Discussion

The present study demonstrates that circuit training at home affects the bone mass as well as other factors, such as the muscle mass and body fat percentage, among undergraduate female students. In the non-exercising group, subjects attained a significantly higher calcaneus QUS-SI at 2 and 3 months from baseline, and in the exercising group, subjects attained a higher calcaneus QUS-SI at 3 months from baseline, albeit not a significantly higher value. These results indicate that subjects in both groups attained a higher bone mass but for different reasons.

Takahata et al. have mentioned that both the body weight and muscle strength affect the QUS-SI in adolescents [9]. Chin et al. observed a relationship between the BMI, QUS-SI, and QUS-BUA in middle-aged and elderly women [26], and Casale et al. showed that the fat-free lean mass had a positive effect on the BMD in pre-menopausal women, while the body fat had a negative effect [27]. This discrepancy in findings may be because of ethnic differences or other differences in subjects’ backgrounds, such as the age, involvement in physical activity, dietary intake, or history of pregnancy.

However, these findings still suggest that both the body fat and muscle mass affect the bone mass. In the present study, exercise and the lack thereof affected the bone mass in different ways; the muscle mass was a significantly positive factor in the exercising group affecting the calcaneus QUS-SOS and calcaneus QUS-BUA, while the body fat percentage was a significantly positive factor in the non-exercising group affecting the calcaneus SI. The body fat percentage was a significantly negative factor in the exercising group affecting the calcaneus QUS-BUA. Lifestyle-related diseases, such as cerebrovascular disorder, often lead to paralysis or death, and overweight is a problem leading to such diseases. Even a short period of exercise seemed to affect the muscle mass and QUS status positively in younger females in the present study, suggesting the importance of instilling an exercise habit at a young age and continuing that the habit to maintain a healthy weight in later life. Casale et al. also mentioned that protein intake during any weight loss interventions is important in order to avoid losing muscle mass [27]. The effects of subjects gaining muscle mass might have been more obvious if the subjects had consumed protein during this study. The present results further showed that it is relatively easy to lose fat mass to obtain a well-proportioned body in a short period with a low-intensity workout in younger females. Heart disease is the second-most common cause of death in Japan, and it is important to maintain a sufficient fat mass with age; therefore, intervention with weight management from a younger age is important and can more easily influence behavior than management introduced at an older age [28]. Sarcopenic obesity is a well-known cause of severe diseases. It is best to instill good exercise habits at a younger age. Several approaches have been suggested for preventing the sarcopenic obesity but the basal metabolism should be maintained by ensuring a sufficient muscle mass [16].

Self-efficacy is important for achieving goals, including health-related goals. Patients suffering from cardiovascular disease or diabetes were able to improve their physical activity through proactive approaches suggested by health specialists in Italy [29]. I did not intervene in the subjects’ performance in the present study aside from performing measurements at the baseline, 2 months later, and 3 months later; however, it would be possible to intervene through a proactive approach to give subjects more opportunities to develop exercise habits. To carry out such studies, it is difficult to approach individuals with no health problems, such as the subjects included in the current study. It is hoped that the current results may motivate healthy subjects to exercise more. In addition, the exercise program described in this study is relatively easy for individuals to continue.

There are several limitations associated with this study. First, I used an ultrasound bone densitometer, a low-invasive technique, to measure the calcaneus QUS-SOS instead of a DXA scan (the preferred technique for measuring the BMD) because the participants were relatively young. Second, the sample size was relatively small. Further research will be needed to confirm how to gain greater bone mass and muscle mass while losing fat mass; the effectiveness of circuit training for improving the calcaneus QUS status, such as QUS-SI, QUS-SOS, and QUS-BUA, as well as building muscle mass in young females. Third, studies involving both sexes may be needed to clarify the sex-based differences in the effect of such exercise on the calcaneus QUS status.

## Conclusions

In conclusion, I observed how circuit training at home can improve the calcaneus QUS status, such as QUS-SI, QUS-SOS, and QUS-BUA, as well as build muscle while losing fat mass through easy sports activities among young females. These findings suggest that circuit training can help females attain significantly greater bone mass and muscle mass and may reduce their risk of osteoporosis or cardiovascular disease in later life. Further longitudinal studies in more subjects are needed in order to gather supporting evidence.
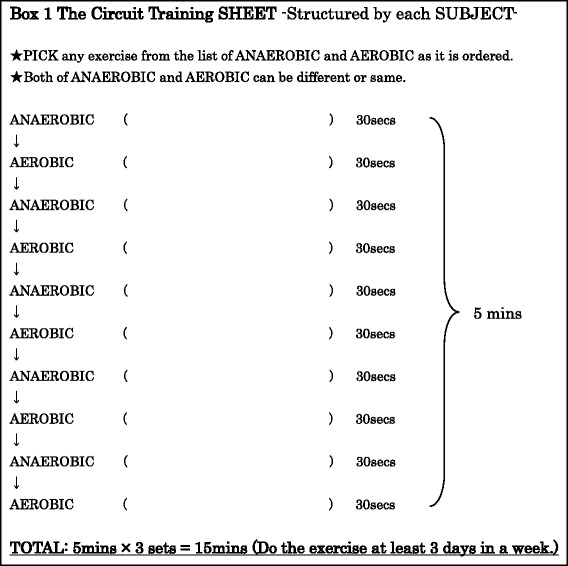


## Abbreviations

BMC: Bone mineral contents
BMD: Bone mineral density
BMI: Body mass index
BUA: Broadband ultrasound attenuation of the calcaneus
DXA: Dual-energy X-ray absorptiometry
QCT: Quantitative computed tomography
QUS: Quantitative ultrasound
SI: Stiffness index of the calcaneus
SOS: Speed of sound of the calcaneus
